# Revisiting Bistability in the Lysis/Lysogeny Circuit of Bacteriophage Lambda

**DOI:** 10.1371/journal.pone.0100876

**Published:** 2014-06-25

**Authors:** Michael Bednarz, Jennifer A. Halliday, Christophe Herman, Ido Golding

**Affiliations:** 1 Department of Physics, University of Illinois, Urbana, Illinois, United States of America; 2 Center for the Physics of Living Cells, University of Illinois, Urbana, Illinois, United States of America; 3 Verna and Marrs McLean Department of Biochemistry and Molecular Biology, Baylor College of Medicine, Houston, Texas, United States of America; 4 Department of Molecular and Human Genetics, Baylor College of Medicine, Houston, Texas, United States of America; 5 Department of Molecular Virology and Microbiology, Baylor College of Medicine, Houston, Texas, United States of America; Tata Institute of Fundamental Research, India

## Abstract

The lysis/lysogeny switch of bacteriophage lambda serves as a paradigm for binary cell fate decision, long-term maintenance of cellular state and stimulus-triggered switching between states. In the literature, the system is often referred to as “bistable.” However, it remains unclear whether this term provides an accurate description or is instead a misnomer. Here we address this question directly. We first quantify transcriptional regulation governing lysogenic maintenance using a single-cell fluorescence reporter. We then use the single-cell data to derive a stochastic theoretical model for the underlying regulatory network. We use the model to predict the steady states of the system and then validate these predictions experimentally. Specifically, a regime of bistability, and the resulting hysteretic behavior, are observed. Beyond the steady states, the theoretical model successfully predicts the kinetics of switching from lysogeny to lysis. Our results show how the physics-inspired concept of bistability can be reliably used to describe cellular phenotype, and how an experimentally-calibrated theoretical model can have accurate predictive power for cell-state switching.

## Introduction

A scenario often encountered in living systems is that of a choice between two alternative cellular fates, driven by different gene expression patterns [1]–[6]. A classical bacterial example is the decision whether or not to utilize a specific carbon source [4], [7]. In metazoans, the eventual fate of a cell is established through a cascade of such binary decision steps during development [2], [3]. Once a cellular state is chosen, the memory of that state can be inheritably maintained [8]–[10]. At the same time, the cell's decision can in some cases be reversed given the proper stimulus (“reprogramming”) [1], [2], [9] or, rarely, be reversed spontaneously [2], [11]–[13].

The life cycle of bacteriophage lambda has long served as a simple paradigm for a binary choice between alternative cell fates and the long-term maintenance of the selected state [1], [5], [14]. Upon infection of an *E. coli* cell, a choice is made between the lytic, virulent pathway that leads to cell death, and the lysogenic, dormant pathway which allows cell survival [1], [15], [16]. Once chosen, the lysogenic state is extremely stable, lasting millions of cell generations under favorable conditions [11], [12]. Lysogeny is not irreversible, however: A lysogenic cell may switch back to the lytic pathway, a process called “induction”. Lysogenic induction can be triggered by a cellular signal (such as DNA damage) [11] but can also occur spontaneously, due to fluctuations in gene expression [11], [12].

**Figure 1:**
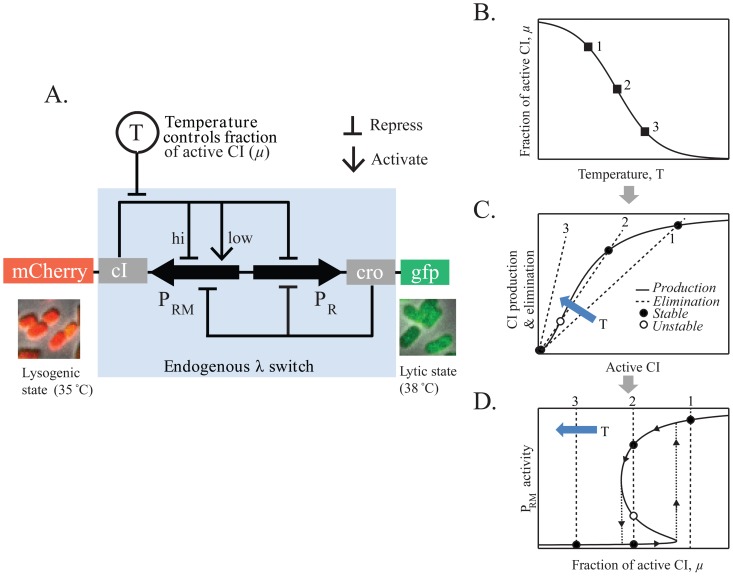
The lysogeny maintenance circuit of bacteriophage lambda. (**A**) A two-color reporter system for measuring P_RM_ and P_R_ activity in individual cells. In the endogenous circuit, cell state is determined by a competition between CI (produced from P_RM_) and Cro (produced from P_R_). After dimerization, both proteins regulate each other's transcription as well as their own. In the reporter system, the *cI* and *cro* transcripts also encode red and green fluorescent proteins, respectively, allowing the detection of P_RM_ and P_R_ activity in individual cells under the microscope. The *cI857* allele used is temperature-sensitive, and temperature is used as a “control knob” in the experiments, by varying the fraction of active CI molecules in the cell. (**B–D**) Using temperature to control cellular state (schematic). At a given temperature, only a fraction *μ*(*T*) of CI molecules in the cell are active (panel **B**). As a result, the balance between CI production and elimination shifts as temperature is changed (panel **C**, plotted as a function of the amount of active CI in the cell). In particular, in the example shown, as temperature increases the system moves from having a single, high-P_RM_ state (corresponding to lysogeny, at temperature #1), to having two stable states (high P_RM_, low P_RM_; bistability, at temperature #2) and finally to a single, low-P_RM_ state (lytic-onset, at temperature #3). Panel **D** depicts the steady states of the system for different temperatures (equivalently, values of *μ*). In the region around #2, hysteretic behavior is expected with cells following the paths indicated by the arrows: Since both states are stable, cells will mostly stay in the state they were originally in. In other words, cell state is dependent on its history.

The lysogenic state is maintained by a single transcription factor, CI (a.k.a. the lambda repressor), produced from the P_RM_ promoter [1] (**Fig. 1A**). As long as enough CI is present in the cell, transcription of the key lytic activator, Cro, is repressed while continuous CI production is maintained through autoregulation [1], [17]. If, however, CI levels drop below a critical level, transcription of *cro* from the P_R_ promoter takes place, leading to halting of CI production and initiation of the lytic pathway [1], [14].

The phenotype exhibited by the lambda system—two distinct cellular fates driven by different gene expression patterns, with one state (lysogeny) very stable but also “switchable”—has led to the usage of the term “bistability” in describing it [18]–[20]. Following the strict mathematical definition [21], [22] bistability means that the two gene-expression states coexist, and each is locally stable, i.e. the system will return to the original state under small perturbations (here, changes in CI level), while a strong enough perturbation will cause the cell to switch from one state to another [23]. Such switch-inducing perturbations can be due to an external source (for example, RecA-driven cleavage of CI [24]), or due to internal fluctuations [12]. In a physical system, such fluctuations would be thermally driven. In living cells, copy-number fluctuations driven by biochemical stochasticity are believed to play an analogous role [12], [25], [26].

The bistability picture is further motivated by theoretical analysis of the gene regulatory circuit governing the maintenance of lysogeny [18], [19], [26], [27]. This analysis takes into account the way *cI* transcription from P_RM_ is modulated by the concentration of CI in the cell, and the balance of CI production and elimination (by degradation or dilution by cell growth). Under plausible choices of model parameters, two stable states are predicted, corresponding to high (lysogeny) and low (lysis) levels of CI (see **Fig. 1C** below) [18], [19], [26].

Following the lambda paradigm, bistability has been invoked for multiple systems exhibiting a binary choice of cell state [20], [28]. In a handful of examples, hysteretic behavior was experimentally demonstrated, providing strong evidence for true bistability (see below) [7], [29], [30]. In most cases, however, the bistability picture was motivated by indirect evidence, such as the presence of positive feedback in the underlying regulatory network; in particular, a self-regulating, fate-determining transcription factor, which is commonly found in systems exhibiting long-term memory of cellular states [2], [8], [9].

In spite of the pivotal role that lambda has played in establishing the bistability paradigm, it is still unclear to what degree this term is actually a proper description of the lysis/lysogeny system, rather than just a loose, and possibly misleading, metaphor. There are multiple reasons to doubt the validity of the bistability description: First, the mere existence of two distinct gene expression states does not immediately imply bistability. Both states simultaneously have to be locally stable for the system to be bistable [28]. In contrast to this situation, some systems (such as the yeast Gal1 promoter) exhibit only a single gene expression state, but that state is modulated in response to external conditions [31]. Second, even if cells in both gene-expression states are simultaneously observed within the population (co-existence), this still does not mean both states are locally stable. A bimodal population can be achieved by other means, such as large but very infrequent bursts of transcription [32], [33]. Third, the fact that one of the lambda states (lysis) is inherently irreversible (leads to cell death) suggests that this state does not necessarily need to be stable to achieve its physiological purpose; instead, even a transient excursion from lysogeny to lysis would suffice for complete switching. This type of “excitable” phenotype has been demonstrated in the competence system of *B. subtilis* [34]. Finally, our current knowledge of gene regulation in the system—how P_RM_ activity depends on CI and Cro amounts in the cell, in particular in the low CI regime—is not quantitative enough to unambiguously determine whether both the lysis and lysogeny states are locally stable.

Thus, to obtain a reliable dynamical-system description of the lysis/lysogeny circuit, and to directly address the bistability question, additional experimental investigation is required. Specifically, one needs to quantify the population structure (i.e. the cell-to-cell CI copy-number statistics) around both the lysogenic and lytic states, as well as the kinetics of switching from one state to another. Most critically, establishing bistability requires demonstrating the existence of *hysteresis*, that is, the history dependence of cellular state. Hysteresis would indicate the presence of two coexisting stable states, with a barrier in transitioning between them. Hysteresis is therefore a necessary consequence of the local stability of both of the lysogenic and lytic states. The existence of hysteresis in the lysis/lysogeny system has been suggested based on colony reporter assays [35]–[37], however a quantitative single-cell study reported its absence [38]. The question of bistability in the lambda system thus remains open.

Here we perform a quantitative characterization of the lambda lysogeny maintenance system using a two-color fluorescent reporter to measure the activity of the P_RM_ and P_R_ promoters in individual cells (**Fig. 1A**). A temperature-sensitive mutant of CI [12], [38]–[40] is used to continuously control the gene-expression state of the cells. We first measure the P_RM_-activity versus CI-concentration regulatory curve and use it to predict the steady states of the system. We find that a regime of bistability is expected. This prediction is then verified experimentally. In particular, we find that hysteretic behavior is exhibited in the expected temperature range. Next, we measure the kinetics of switching from lysogeny to lysis. The observed kinetics again show good agreement with the theoretical prediction based on our measured parameters of stochastic gene expression in the system. Our findings thus validate the bistability attribution of the lambda lysis/lysogeny circuit and provide a quantitative framework for describing the system dynamics.

## Results

### A two-color reporter for transcriptional activity in the lysogeny maintenance circuit

For the purpose of this study, we constructed a two-color reporter strain that allows us to quantify simultaneously, in individual cells, the activity of the two key promoters in the lysogeny maintenance circuit: P_RM_, from which *cI* is transcribed, and P_R_, from which *cro* is transcribed (**Fig. 1A**). In constructing this reporter, we followed the approach of [41]. The region of the lambda genome governing the maintenance of lysogeny (position 37112–38458 bp) [41], was inserted into the *lac* locus of the *E. coli* chromosome (strain MG1655). This segment of the phage genome includes the genes *cI* and *cro* as well as the three operator sites (O_R_1-3) where CI and Cro bind and regulate the expression from P_RM_ and P_R_ [1] (The distant O_L_1-3 operator sites are not included, see **Discussion** below). For reporting on the transcriptional activities of P_RM_ and P_R_, genes encoding green (GFP) and red (mCherry) fluorescent proteins were fused to *cro* and *cI*, respectively, creating transcriptional fusions. Thus, CI and mCherry are produced from the same transcript, and similarly Cro and GFP are produced from the same transcript (**Fig. 1A**). By encompassing the complete regulatory circuit governing the maintenance of lysogeny, the reporter system supports the two fundamental states of the system: a high CI/low Cro (red cells) state, corresponding to the lysogenic state of the full phage; and a low CI/high Cro (green cells) state, corresponding to the onset of lysis (**Fig. 1A**). (Unlike a true lysogen, however, the reporter strain does not contain the full viral genome and therefore actual lysis does not ensue in the latter case [41]. For simplicity, however, we refer to the low CI/high Cro state as “lytic”.)

The use of temperature as a control parameter in our system is depicted schematically in **Fig. 1B-D**. The *cI* allele used in our reporter system is a temperature-sensitive mutant, *cI857* [12], [38]–[40]. At the permissive temperature (*T* ⪝ 32°C), CI857 behaves similarly to wild-type CI [12], [38], [40]. As temperature is increased, however, an increasing fraction of CI proteins in the cell become non-functional [12], [42]. The temperature-dependent fraction of active CI molecules, *μ*(*T*) (**Fig. 1B**), governs the strength of feedback that CI exerts on the P_RM_ promoter [21], see **Text S1** for more details. The result is that, by varying the temperature we can continuously change the balance of CI production and elimination (**Fig. 1C**, plotted as a function of the amount of active CI in the cell) and thus tilt the balance between the lysogenic and lytic states [12], [38]. Depending on the precise way in which P_RM_ activity depends on CI concentration, three different regimes may be encountered as temperature is changed (**Fig. 1D**): One, where only the lysogenic state is stable (high CI expression, marked #1); another, in which only the lytic state (low CI expression) is stable (#3); and in between, there may be a range of temperatures where both the lysogenic state and lytic state are stable (#2), that is, bistability is supported. The key to determining the steady states of the system, in particular the possible existence of bistability, is to be able to describe quantitatively the balance of CI production and elimination. To that end, we first characterize in more detail the regulation of P_RM_ activity by CI.

### Measuring the P_RM_(CI) regulatory curve

**Figure 3:**
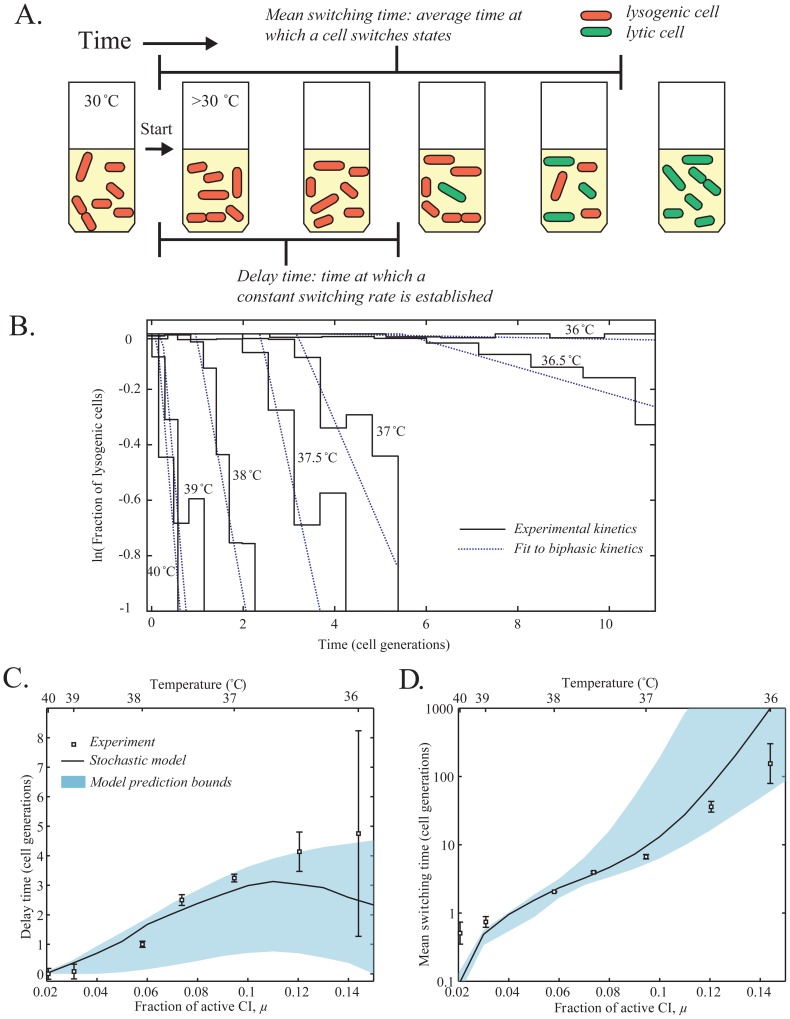
The kinetics of switching from lysogeny to lysis. (**A**) A schematic of the switching experiment. Cells were initially grown at 30°C for ∼5 generations, and then moved to a higher temperature. As a result, the population gradually switched from “red” (high CI, lysogenic) to “green” (high Cro, lytic). Two variables were extracted: The time when switching at a constant rate begins (designated the delay time) and the average time at which a cell switches from lysogeny to lysis (designated the mean switching time). (**B**) The fraction of remaining lysogens as a function of time, for different end temperatures in the range 36–40°C. Each stair-step graph shows experimental data from a corresponding experiment. The dashed line is a fit to the observed biphasic behavior: delay followed by exponential switching. The fit was used to estimate the delay time and the mean switching time. (**C**) The delay time before switching as a function of the fraction of active CI, *μ* (equivalently, temperature). Squares, experimental results and standard error from 3 independent experiments. Solid line and shaded region, results of the stochastic model and their estimated error. (**D**) The mean switching time as a function of the fraction of active CI, *μ* (equivalently, temperature). Notation as in panel **C**.

Recall that CI is produced from the P_RM_ promoter, whose transcriptional activity is in turn regulated by the binding of CI dimers to the O_R_1-3 operators [1]. We set out to quantify the autoregulatory response curve *f*(*x*), which describes the activity of P_RM_ (*f*, number of proteins produced per generation time) as a function of CI concentration in the cell (*x*, number of active CI molecules per cell). Note that the regulatory effect of Cro, which also regulates P_RM_ [1], [37], is only included implicitly here: Cro's presence when CI levels are low leads to a strong repression of P_RM_ in that regime [37]. This one-dimensional simplification may be justified by understanding that, at a given CI concentration, Cro will always adopt a single steady-state value, i.e. the steady-state level of Cro is a function of CI only (Cro is also subject to negative autoregulation [21], [37]). This implies that the steady-state P_RM_ promoter activity can be written entirely as a function of CI level. Despite its simplicity, this “one-dimensional” approximation remains sufficient when extended to predicting the *kinetics* of the system (see **Fig. 3C–D** below).

To estimate *f*(*x*), we first note that the requirement for a steady-state, namely a balance between protein production and elimination, leads to two important relations (see **Text S1** for more details): First, *f*(*x*) α *F*(*T*), where *F*(*T*) is the mean cell fluorescence at a given temperature. In other words, at each temperature, the mean cell fluorescence is proportional to P_RM_ activity at the steady-state CI level at that temperature (this proportionality constant may be set to 1 by measuring both fluorescence and P_RM_ activity relative to their maximum values). Second, x = *μ*(*T*) X *F*(*T*), that is, the concentration of active CI in the cell, *x*, is given by the product of the mean fluorescence and *μ*(*T*), which describes the fraction of CI molecules that are active at temperature T [12](**Fig. 1B** above). Thus, knowing *μ*(*T*) allows us to estimate simultaneously the amount of CI and the resulting P_RM_ activity at a given temperature. *μ*(*T*) (or an equivalent parameter) has been estimated in a number of studies [12], [38], [40]. Here we follow [38] and use the approximation that *μ*(*T*) = *γ*(*T*)/(*γ*(*T*)+*e* ^k(T-32OC)^) with *k = 0.55* (as in [38]) and *γ*(*T*) the growth rate at temperature *T* (normalized to maximum growth rate seen in experiments: doubling time of ≈28 minutes at 40°C). We note that using the (slightly different) expressions from other studies [12], [38], [40] leads to similar results in our analysis below (**Table S1**).

**Figure 2:**
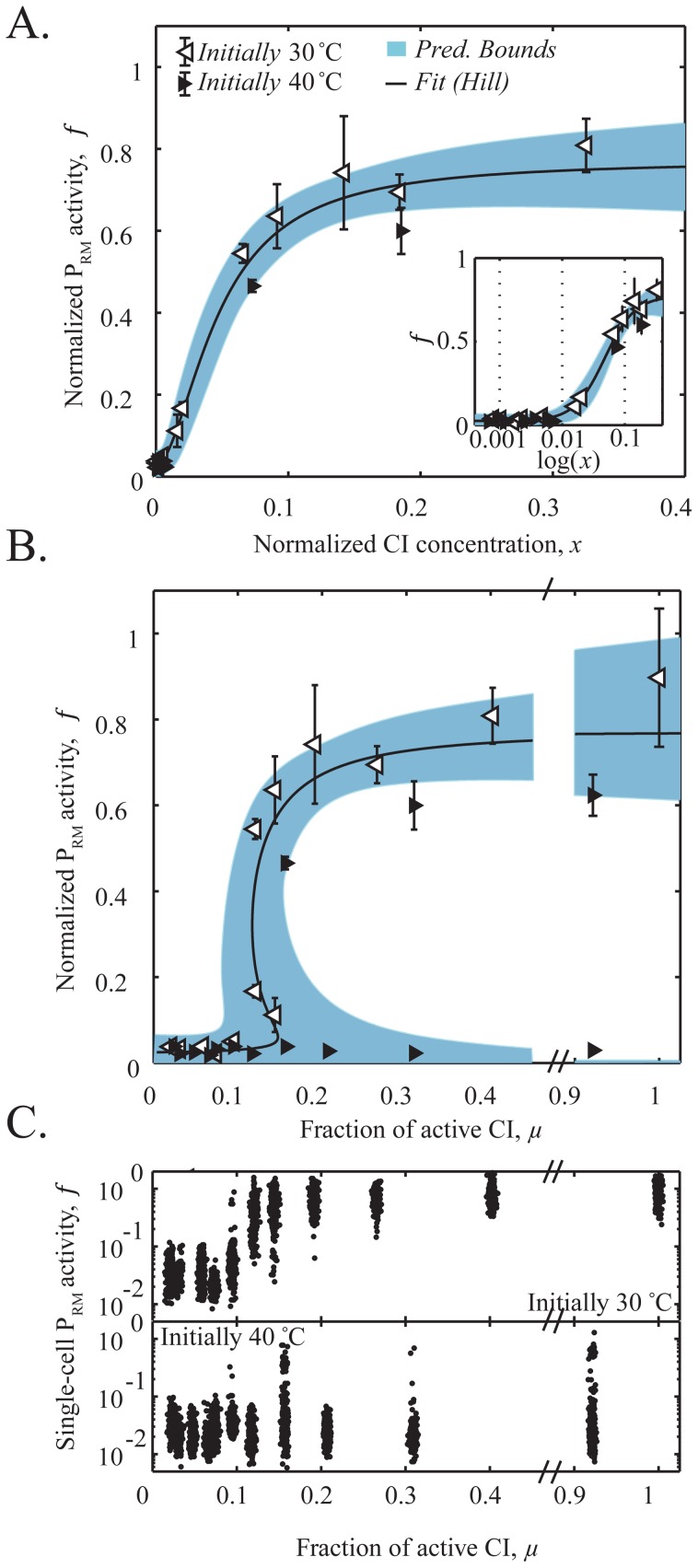
Experimental demonstration of bistability and hysteresis. (**A**) P_RM_ promoter activity as a function of CI concentration in the cell. Both observables were measured as described in the main text, and are normalized by the mean promoter activity of the initial lysogenic state (at 30°C). Black and white triangles represent data from cells initially grown in the lytic (40°C) and lysogenic (30°C) states, respectively. Each data point was obtained by averaging over three independent experiments. Error bars represent the standard error of these three experiments. The solid line is a fit to a Hill function and the shaded blue area is the 95% confidence interval of that fit. The inset depicts the same data in semi-logarithmic scale. (**B**) Predicted and measured steady-states of the lysis/lysogeny circuit. Prediction: Solid line, predicted P_RM_ steady states based on the fitted P_RM_ regulatory curve in panel A. Shaded region represents the confidence boundary of the theoretical prediction. Measurement: white and black triangles are the same data set depicted in panel A, plotted with respect to *μ*. (**C**) Hysteretic behavior of the lysis/lysogeny circuit. The P_RM_ activity of individual cells is plotted, for cultures grown initially at low temperature (high P_RM_ activity, **top panel**) and cultures grown initially at high temperature (low P_RM_ activity, **bottom panel**). Each dot represents one cell (total 300 cells randomly chosen from our data at each temperature). For visualization purposes, individual data points were given a small random horizontal deviation, centered on the correct value of *μ* for that temperature.

Using the relations above, we measured *f*(*x*) as follows. Cells were grown at different temperatures in the range 34–40°C (corresponding to *μ*≈0.4–0.02). At each temperature, we measured the mean population fluorescence during exponential growth, *F*(*T*). When two distinct populations were present (see **Fig. 2C**, **Fig. S1** in **Text S1**), the mean fluorescence for each sub-population was estimated separately. *F*(*T*), combined with *μ*(*T*), allowed us to estimate P_RM_ activity *f* as a function of active CI concentration *x*. The resulting estimate is depicted in **Fig. 2A**. The curve exhibits the expected features based on the known properties of system: At CI≈0, P_RM_ has a low level of basal activity [37]. As CI levels increase, P_RM_ displays a cooperative (super-linear) increase in activity [17], [18], [37], [43] until a maximal activity is reached. Experimentally, *f*(*x*) is well approximated by a Hill function [4], *f*(*x*) = *f*_0_ {*ε*+ (1−*ε*)/(1+ [*x*/*x*_0_]^−*H*^)}, with *f*_0_  = 0.77±0.08, *ε* = 0.033±0.032, *H* = 1.80±0.57, *x*_0_ = 0.062±0.011. The estimated Hill coefficient *H* and basal activity *ε* are in good agreement with previous population-based studies [37], (see **Fig. S2** in **Text S1**). The shaded region in **Fig. 2A** represents the uncertainty in the fit (see **Text S1**).

### Bistability and hysteresis

The measured autoregulatory response-curve *f*(*x*) was next used to predict what the steady states of the system are as we vary our “control parameter”, temperature. To do so, we followed the approach of [22] and plotted the fitted *f*(*x*) versus *x*/*f* (which, at steady state, is equal to *μ*(*T*)) (**Fig. 2B**). This plot directly provides the predicted steady-state (or states) of a system whose production kinetics are governed by *f*(*x*), as a function of the fraction of active CI, *μ*(*x*). We also plot (blue shaded area) the estimated error in predicting the steady states (see **Text S1**) based on the uncertainty in the fitting parameters of the P_RM_ response-curve *f*(*x*) (A similar error analysis was performed for the possible pleiotropic effects of changing the growth temperature, see **Fig. S3** in **Text S1**). It can be seen that in the range *μ*≈0.12–0.15 (approximately 35.5°C to 36.5°C), two stable states are predicted. The error analysis extends this possible bistability range from *μ*≈0.08 all the way to *μ*≈1, indicating that the lysogeny maintenance circuit in a wild type phage may support bistability.

An important consequence of the predicted bistability is that the system is expected to display a hysteretic behavior [22]; that is, the gene-expression state of a cell will depend on its history, not just on the conditions at the time of measurement. Specifically, if we start with a population of cells at low temperature, all in the lysogenic state, and shift to a higher temperature, then, if in that higher temperature both lytic and lysogenic states are stable, most cells will still remain in the original lysogenic state, due its local stability to perturbations. But once the temperature has been increased sufficiently, such that the original lysogenic state is no longer stable, the population will all switch to the alternative (and now the only stable) state, lysis. When this procedure is repeated in a reverse manner, starting at high temperature and shifting to a lower temperature, the opposite trend will be observed: most cells will remain in the lytic state until “forced” into lysogeny when lysis is no longer stable. The combined result is that the state of the system cannot be predicted from the current temperature, but instead depends on the history—at what temperature the cells were grown previously [22].

We next turned to test these theoretical predictions experimentally. To do so, cells were first grown overnight at a temperature that supported a uniform P_RM_ state, either lysogenic (30°C) or lytic (40°C). The cultures were diluted 10^6^ fold and grown at different temperatures in the range 34–40°C for >15 cell generations (8–14 hours) while maintaining exponential growth. P_RM_ activity in ∼300 individual cells was then determined based on their red fluorescence, and the distributions of single-cell P_RM_ activities were examined (**Fig. 2C** and **Fig. S1** in **Text S1**). The observed histograms consisted of either one or two peaks, corresponding to the lysogenic (high P_RM_ activity) or lytic (low P_RM_ activity) states. As seen in **Fig. 2B**, P_RM_ activities at each temperature (black and white triangles) were in good agreement with the theoretical prediction (solid curve and shaded area). In addition, the expected hysteretic behavior was observed (**Fig. 2C**): In cultures that were initially grown at 30°C, the majority of cells (>90%) remained in the lysogenic state when grown at temperatures as high as 36°C (*μ*≈0.12), despite the fact that the lytic state is already stable at this temperature. Similarly, in cultures that were initially grown at 40°C, most cells remained lytic (>90%) even when grown at 30°C (*μ*≈1) (despite the stability of lysogeny at temperatures <36.5°C. Thus, bistability is present in the temperature range 30–36°C (*μ*≈0.12–1), in agreement with the theoretical prediction (within the uncertainty bounds of our measurements) based on the measured P_RM_(CI) activity curve. In particular, since CI857 is fully functional at 30–32°C [12], [38], [40], we interpret our data to mean that the lysogeny maintenance circuit of wild-type phage exhibits bistability as well, i.e. both the high-CI (lysogeny) and high-Cro (lytic onset) states are stable.

### The kinetics of switching from lysogeny to lysis

After characterizing the steady states of the system, we next turned to examining the kinetics of switching from one state to another. In particular, we wanted to further test the validity of our theoretical description by asking whether it allows us to predict the quantitative features of switching from lysogeny to lysis.

Experimentally, switching was induced by initially culturing cells at 30°C (lysogenic state) for ∼5 generations and then transferring the culture to a different temperature in the range of 36–40°C. The cells were then grown for 2.5–11 hours (depending on temperature) and their state tracked by imaging ∼100 cells from samples taken every 15–60 minutes (shown schematically in **Fig. 3A**). The state of each cell was determined by measuring both P_RM_ (red) and P_R_ (green) signals, and the fraction of cells that have switched to the lytic state was determined by setting a threshold of green to red fluorescence (**Fig. S4** in **Text S1**). The observed switching kinetics are depicted in **Fig. 3B**. It can be seen that switching from lysogeny to lysis did not proceed immediately after the temperature shift, but rather following a delay. This delay depended on the end temperature (**Fig. 3C**), increasing up to >4 generation times for a shift to 36°C. Control experiments demonstrated that the observed delay does not reflect the fluorescent proteins' maturation time (**Fig. S5** in **Text S1**). Following the delay period, state switching proceeded at a constant rate, such that the fraction of cells remaining in the lysogenic state declined exponentially in time (**Fig. 3B**). The switching rate (or equivalently, the mean switching time), too, depended on the end temperature (**Fig. 3D**).

To obtain a theoretical prediction of switching kinetics, we needed to characterize not only the steady states of the system (as done above) but also the stochastic kinetics of protein production, which drive the transition between states [12], [26], [27], [44]. These kinetics can in turn be inferred from examining the statistics of protein copy-numbers at steady state [45]–[47]. In our reporter system, the histograms of P_RM_ activity (**Fig. S1** in **Text S1**) consisted of one or two peaks; each peak was well described by a gamma distribution [45]. This is consistent with a scenario in which CI is produced in random bursts [12], [48], [49], where both burst size and inter-burst intervals are exponentially distributed [12], [48], [50], [51]. The measured mean and variance of the fluorescence distributions (see **Fig. S6B** in **Text S1**), when combined with the estimated number of CI proteins per cell in the high-CI state (∼300, [52]–[56]), allowed us to directly estimate both the frequency of bursts (or probability per unit time of having a burst), *a*, and the average number of proteins produced in each burst (burst size), b, at a given promoter activity level (**Fig. S6C** in **Text S1**). We found that both parameters can be approximated using a simple power-law dependence on the mean CI expression level: *b* = [*f*/*φ*]*^δ^*, *a* =  *φ*[*f*/*φ*]^1−*δ*^, with *δ* = 0.68±0.14 and *φ = 5.3*±3.5 generation^−1^ where *f* is again the promoter activity measured by the mean fluorescence in the cell. These simple relationships are similar to those found for other genes in *E. coli* [12], [57] as well as in yeast [58] (see **Text S1**). The extracted parameters above were next incorporated into a stochastic model of the lysis/lysogeny regulatory circuit (**Fig. S6A** in **Text S1**) and used to predict single-cell CI kinetics when switched from one temperature to another. In our model, CI is produced stochastically from P_RM_. The activity level of P_RM_ is determined by the instantaneous concentration of CI in the cell, using the P_RM_(CI) relation obtained above. Each P_RM_ activity level is manifested in a specific frequency (*a*) and average size (*b*) of CI production bursts, estimated as described in the previous paragraph. CI is also diluted at the rate of cell growth [12], [18], [19], [26]. To solve this stochastic model, we wrote down the master equation describing the temporal evolution of the probability distribution of CI numbers, *p*(*n*, *t*) [59], see **Text S1**. This equation was then numerically solved using the Padé approximation [60], [61]. Doing so allowed us to obtain the full population statistics of CI numbers at any given time point.

To model a switching experiment, the cell population was first equilibrated for 100 generations in the low temperature limit (*μ*(*T*) = 1, i.e. T≈30°C), and then shifted into a higher temperature, represented by the corresponding *μ*(*T*)<1. The probability distribution of CI numbers was followed for 12 cell generations. At each time point, the fraction of “switched” cells was estimated by calculating the probability of having CI numbers below a threshold corresponding to the value found in experiments (**Fig. S4** in **Text S1**). This procedure was performed for *μ* = 0.15–0.01 (Temperatures ∼35.5–41°C) simulating the switching experiment above. When the predicted switching kinetics (fraction of lysogenic cells as a function of time) was compared to the experimental data (**Fig. 3B–D**), good agreement was observed. The stochastic model, like the experimental data, shows a delay period followed by switching at a constant rate (**Fig. S7B** in **Text S1**). Furthermore, the model succeeds in predicting both the delay period (**Fig. 3C**) and the mean switching time, i.e. the average time at which a cell switches from lysogeny to lysis (**Fig. 3D**). Thus, the measured P_RM_(CI) regulatory curve, when combined with the measured parameters of stochastic CI production, is successful in predicting not only the steady states of the system but also the kinetics of switching from one state to another.

## Discussion

Our observation of bistability in the lambda lysis/lysogeny system extends and complements previous works that have pointed in that direction: (i) Plate-based reporter studies, showing that colonies in the “anti-immune” (high Cro, low CI) rarely switch back to “immunity” (lysogeny) even after long incubation at a lower temperature, thus suggesting that the Cro-dominated state was inheritably stable [35]–[37]. (ii) Theoretical work suggesting that the CI/Cro regulatory circuit can lead to the emergence of two stable states, corresponding to lysis and lysogeny [18], [19]. Here, we demonstrated for the first time a quantitative agreement between the theoretical and experimental descriptions of bistability in the lambda system. Specifically, we showed that the experimentally measured P_RM_(CI) regulatory curve allows us to successfully predict the stable states of the system. A regime of bistability was predicted, and then confirmed experimentally via the presence of hysteretic behavior. Importantly, we also found that the hysteretic behavior extended to temperatures as low as 30°C: A culture of “lytic” cells (cultured overnight at 40°C) largely remained in the lytic state (expressing Cro) after growth at 30°C for over 15 cell generations. At these temperatures, our temperature-sensitive allele is fully functional [12], [38], [40]. Thus, the data strongly suggests that wild-type lambda lysogens support bistability, i.e. both the high-CI (lysogeny) and high-Cro (lytic onset) states are stable. The implication is that the lytic-onset state is not easily reversed: Once a large-enough excursion away from lysogeny occurs, the cell becomes committed to the lytic fate [37], [41].

In contrast to our findings here, a previous single-cell study of the lysis/lysogeny switch [38] did not find evidence for hysteresis. We attribute the lack of hysteresis to the absence of the *cro* gene in the reporter system used in [38]. P_RM_ repression by Cro has been suggested to play a critical role in stabilizing the lytic-onset state [37]. Indeed, our theoretical analysis of the P_RM_(CI) curve measured in the absence of Cro by [37] suggests that no bistability is expected in that case (**Fig. S2** in **Text S1**).

From a functional perspective, the stability of both alternative states presumably renders the cell's decision irreversible—or at least not easily reversible. Our observation of bistability in the lambda lysis/lysogeny switch complements earlier findings of hysteresis in other regulatory networks [7], [29], [30], where limiting reversibility may be advantageous.

After validating the prediction of the stable states, we used the measured P_RM_(CI), in conjunction with the estimated parameters of stochastic CI production, to successfully predict the kinetics of switching from lysogeny to lysis. When shifted from one temperature to another, a population of cells exhibited delayed switching, with both the delay period and the switching rate dependent on the new temperature. This “biphasic” behavior can be understood as follows: During the delay period, CI distribution in the population relaxes to a new form following the shift of balance between production and dilution (due to temperature change). Next, a constant switching rate is established, representing the “leakage” of cells out of the locally-stable potential well [23], [26], [44]. In accordance with this picture, the switching time (or equivalently, the switching rate) is exponentially sensitive to the value of feedback strength *μ*(*T*). This exponential dependence also conforms to a previous study of lysogen stability [12].

Despite our success in linking the theoretical and experimental descriptions of system dynamics, a few caveats are in place. A first one has to do with our use of a temperature-sensitive mutant (*cI857*) for controlling the state of the system. This allele has been successfully used in quantitative studies in the past [12], [38], [40]. Nevertheless, when using temperature as the “control knob”, one has to be wary of possible pleiotropic effects due to the change in rates of cellular processes as temperature is modified [62], [63]. To control for such effects, we estimated the expected error in measuring P_RM_(CI) and the resulting steady states, due to possible temperature-related changes in transcription and translation rates (**Text S1**). As seen in **Fig. S3** in **Text S1**, temperature effects are not expected to have a significant effect on the essential dynamic features of the lysis/lysogeny system.

Second, we note that the absence of the O_L_1-3 operator sites in our construct limits the repression of P_RM_ at high CI levels, compared to what is seen in the wild type system [17], [64], [65] (**Fig. S2A** in **Text S1**). However, the resulting change in P_RM_(CI) is not expected to affect the number of stable states exhibited by the system, merely change the P_RM_ steady-state level at the lysogenic state. In agreement with this expectation, we found a quantitative agreement between the derived steady-states of [37], in which the O_L_1-3 operator sites are present, and our experimental results with the O_L_^−^ reporter strain (see **Fig. S2B** in **Text S1**). Specifically, the regimes of bistability are comparable, indicating that our prediction of bistability would be maintained in the presence of the O_L_ operator.

Finally, in this work state-switching was not quantified in individual cells over time, due to the technical limitations of following single-cell fluorescence over long durations under the microscope [66]. Instead, the population structure (in terms of P_RM_ and P_R_ activity) under different conditions was obtained by taking “snapshots” of many individual cells at different time points. This approach proved very successful in our effort to characterize hysteretic behavior and switching kinetics in the system. Nevertheless, once the technical obstacles are overcome, a promising future direction will be to investigate how the dynamic maintenance of cellular state, and the switching between states, look like when examined in real time in individual cells. Directly observing the how a single cell “climbs out” of lysogeny, against the pull of the P_RM_(CI) regulatory feedback, and then “falls into” the lytic state, will be an exciting next step in forming our quantitative narrative for the lambda system.

## Materials and Methods

### Construction of reporter strain CH1354

Recombineering was performed using the method of [67]. The genes encoding mCherry and Chloramphenicol resistance (Cat) were recombineered into strain NC414 (W3110 lac^0^<>*kan-Ter*<>*luc*<>*rexA cI857 pRORcro^+^cII* <>*lacZYA* [41]), upstream of *cI-cro-lacZYA.* The resulting strain, CH1344, was used to create a P1 phage lysate and transduce the mCherry-Cat region into strain CH1118 (MG1655 *lacZYA::GFP::FRT*).

### Cell culturing and sample preparation for hysteresis experiments

Cells were grown overnight in LB [68] at either 30°C (lysogenic) or 40°C (lytic). Cells were then diluted 10^6^ fold into 1 ml M9 medium (Teknova M8010) containing glucose (2%) and casamino acids (0.01%) and grown in shakers at intermediate temperatures as described in the text. Cells were grown to an optical density (OD600) of ∼0.2 at which time they were pelleted by centrifugation and resuspended in 100 µl of PBS (Teknova P0200). Cells were then stored at 4°C until microscopy was performed (2–8 hours after resuspension).

### Cell culturing and sample preparation for switching kinetics experiments

Cells were grown overnight in LB at 30°C (lysogenic). Cells were then diluted 10^3^ fold and grown in 25 ml M9 medium containing glucose (2%) and casamino acids (0.01%) at 30°C. When cells reached an OD600∼0.1 the cells were shifted to another shaker at a higher temperature (36–40°C). Immediately prior to the transfer, a 1 ml sample of culture was removed, pelleted by centrifugation and resuspended in 100 µl of PBS. Cells were then stored at 4°C until microscopy was performed (2–8 hours after resuspension). After the transfer, additional 1 ml samples were taken at regular intervals and the same procedure performed. At each time-point the optical density was measured, when OD600 exceeded ∼0.4 the culture was diluted 2–4 fold (depending on growth rate and time between data points) so that the culture constantly remained in log-phase.

### Microscopy

All microscopy was performed by placing 1–2 µl of resuspended cell culture between a 1.5% PBS Agar (∼1 mm thick slice) and a glass coverslip (no. 1; Fisher Scientific). Images were acquired using an inverted epifluorescence microscope (Eclipse TE2000-E, Nikon) and cooled CCD camera (Cascade 512, Photometrics). An 100x oil immersion objective was used in concert with an x2.5 coupling lens ahead of the camera. Data from 3 channels were collected. Phase contrast images were collected from 11 z-positions at 100 nm spacing with a 100 ms exposure time. Fluorescence images using the TexasRed (Nikon) and GFP (Nikon) filter sets were taken at the central z-position with 500 and 1600 ms exposure times respectively.

### Image analysis

Cell recognition was performed on the best phase contrast image in the z-stack using the *Schnitzcell* program [69] (gift of Michael Elowitz, Caltech), and the mean fluorescence level (after the subtraction of background) in the recognized cells was measured.

### State determination

The ratio of GFP fluorescence to total fluorescence was used to set a criterion for whether a cell was in the lysogenic or lytic state (**Fig. S4** in **Text S1**). This criterion is equivalent to setting an angle in the GFP vs. mCherry plane as the partition between lysogenic and lytic cells.

## Supporting Information

Text S1 This file contains the following additional data: Use of the temperature-sensitive allele; predicting the steady-states of the lambda switch; error propagation; estimation of burst parameters from single-cell data; the stochastic model for the lambda switch; estimating pleotropic effects of changes in temperature; **Table S1**, The effect of using different models for the effect of temperature in the *cI857* allele; **Figure S1**, Single-cell distributions of P_RM_ Activity at different temperatures; **Figure S2**, Comparison of our results with those of (Schubert et al., 2007); **Figure S3**, Estimating pleotropic effects of changes in temperature; **Figure S4**, Defining the lysis/lysogeny threshold; **Figure S5**, Fluorescence maturation kinetics; **Figure S6**, Defining the parameters of the stochastic model; **Figure S7**, Results of Stochastic Model.
(PDF)
