# Supplementary material for: Revisiting Bistability in the Lysis/Lysogeny Circuit of Bacteriophage Lambda
Source: PLoS One. 2014 Jun 25;9(6):e100876. doi: 10.1371/journal.pone.0100876 (PMC4070997; doi:10.1371/journal.pone.0100876)
Supplement: Text S1 — This file contains the following additional data: Use of the temperature-sensitive allele; predicting the steady-states of the lambda switch; error propagation; estimation of burst parameters from single-cell data; the stochastic model for the lambda switch; estimating pleotropic effects of changes in temperature; Table S1, The effect of using different models for the effect of temperature in the cI857 allele; Figure S1, Single-cell distributions of PRM Activity at different temperatures; Figure S2, Comparison of our results with those of (Schubert et al., 2007); Figure S3, Estimating pleotropic effects of changes in temperature; Figure S4, Defining the lysis/lysogeny threshold; Figure S5, Fluorescence maturation kinetics; Figure S6, Defining the parameters of the stochastic model; Figure S7, Results of Stochastic Model. (PDF) [file pone.0100876.s001.pdf]

TEXT S1

Use of the temperature-sensitive allele: To tune CI protein level in the cell, we use the temperature-sensitive allele *cI857* (Hershey, 1971). In this mutant, CI bears a GCA to ACA mutation at bp 199 (Lieb, 1981). The precise way by which temperature dependence arises in this mutant is not known. Phenomenologically, two alternative scenarios have been invoked to describe the effect of temperature on CI in the cell: (1) active degradation of CI protein, at a temperature-dependent rate $d(T)$ (Isaacs *et al*, 2003; Villaverde *et al*, 1993); (2) loss of functionality, such that only a fraction $\mu(T)$ of CI proteins is active, i.e. able to bind DNA and regulate transcription (Zong *et al*, 2010; Gaitanaris *et al*, 1994). Importantly, both scenarios result in the same steady-state promoter activity levels if the following mapping is made: $\mu(T) = \gamma(T)/(\gamma(T) + d(T))$, where γ is the growth rate. In this work, we assume the second scenario, i.e. that the effect of temperature is to change the fraction of active CI molecules in the cell. This choice is motivated by three considerations. First, CI857 has been found to be capable of renaturing in the cell (Gaitanaris *et al*, 1994), an observation inconsistent with irreversible degradation. Second, a model assuming a temperature-dependent fraction of active CI successfully predicts the stability of *cI857* lysogens at different temperatures (Zong *et al*, 2010). Third, the increased degradation scenario was incapable of quantitatively reproducing the delayed switching kinetics observed in our experiments (**Figure 3B-D**).

Predicting the steady-states of the lambda switch: The steady-states are defined by the requirement that CI production is balanced by CI loss through dilution

$$\frac{dx}{dt} = f(x) - x = 0 \qquad (1)$$

where $f(x)$ is P_{RM} activity (the amount of CI produced per generation time) and x is the mean concentration of CI molecules per cell. The rate of loss is 1, since units are chosen to be generation times (cell interdivision time/ln(2)).

In our reporter strain, equation (1) must be modified due to the fact that only a fraction of CI proteins, $\mu(T)$, is active.

$$\frac{dx}{dt} = \mu(T) f(x) - x = 0 \qquad (2)$$

Where we have used the transformation $x \to \mu x$ so that x now represents the concentration of *active* CI molecules. This expression may be rewritten:

$$x = \mu(T) f(x) \qquad (3)$$

An additional feature of our strain is the presence of the fluorescent reporter. Its steady-state is defined by the expression:

$\frac{dF}{dt} = F_0 f(x) - F = 0$ (4)

where F is the cell fluorescence and F_0 is a normalization constant. We can therefore equate $F = f(x)$ at steady-state using the appropriate normalization.

We can also rewrite equation (3) as:

$x(T) = \mu(T)F(T)$ (5)

This last relation allows us to construct the regulatory curve of the P_{RM} promoter with respect to CI from experimental fluorescence data, i.e. plot $f(x)$ vs. x as we do in **Fig. 2A**. Conversely, given a theoretical fit for the dose response function $f_{TH}(x)$ we can obtain a theoretical prediction of the steady-states of the system $f_{TH}(x)$ vs. $\mu(x)$, since, at steady-state,

$\mu(x) = x/f_{TH}(x)$ (6)

This theoretical curve is shown as the solid line in **Fig. 2B**, where we have plotted the predicted steady-states using the theoretical fit for the dose-response obtained in **Fig. 2A.**

Error propagation: To propagate errors resulting from the fitting of experimental data, we first calculated the prediction bounds and confidence intervals for the fit of the P_{RM} versus active CI data (**Fig. 2A**) to a Hill function, and for the fit of P_{RM} activity variance versus mean to a power law (**Fig. S6B**,). This was done using Matlab's curve fitting toolbox. The prediction bounds represent the inference from the data that the true functional value of the fit lies within the prediction bounds with 95% certainty. To then estimate the prediction bounds for the P_{RM} steady-states, the individual functions representing the upper and lower bounds of the P_{RM}(CI) curve were transformed analogously to the nominal fit.

To estimate the parameter bounds for the stochastic model (see Supplemental Information Below), 100 random parameter sets were chosen from within the confidence intervals of both experimental fits above, subject to the further requirement that the mean standard error (MSE) of the fit be less than twice that of the fit given by Matlab's curve fitting algorithm. This requirement was imposed in order to closely match the prediction bounds of the individual fits. The prediction bounds for the delay time and mean switching time in our stochastic model represent the minimum and maximum values of those observables obtained through this random sampling.

Estimation of burst parameters from single-cell data: The mean protein burst size $b(x)$ and burst frequency $a(x)$ were extracted from experimental data by calculating the dependence of the cell-to-cell variance σ^2 in P_{RM} activity on the mean activity f. This was done as follows: the single-cell fluorescence data

from experiments at all temperatures (**Fig. 2C**) were combined, and then binned based on the level of active CI x. The variance and mean of red cell fluorescence were then approximated using a power law relation, $\sigma^2 = [\frac{f}{\varphi}]^{1+\delta}$ (**Fig. S6B**), with $\delta = 0.68 \pm 0.14$ and $\varphi = 5.3 \pm 3.5\ generation^{-1}$ in molecular units (*see below*). This allowed us to estimate the burst size and frequency for a given level of x, using the relation $a = \frac{f^2}{\sigma^2}$ and $b = \frac{\sigma^2}{f}$, (Shahrezaei & Swain, 2008; Friedman *et al*, 2006; Mehta *et al*, 2008) (**Fig. S6C**). The parameter φ was rescaled from fluorescence units to molecule number, using the estimated value of ~300 total CI molecules/cell (Levine *et al*, 1979; Reichardt & Kaiser, 1971).

A stochastic model for the lambda switch: We assume that the switching between lysogeny and lysis is governed by the stochastic kinetics of CI copy numbers. We therefore model these kinetics according to three assumptions (shown schematically in **Fig. S6A**):

1. CI is produced from P_{RM} in geometrically distributed bursts of mean size b and frequency per generation a, both of which are functions of the instantaneous level of active CI (see **Estimation of burst parameters from single-cell data**).
2. CI is diluted by cell division.
3. The onset of lysis occurs once CI level in the cell drops below a certain threshold.

These assumptions can be captured using a master equation, which specifies how the population structure (in terms of CI copy number) evolves in time (Kampen, 1981; Mehta *et al*, 2008). Specifically, the master equation is written as: $\frac{dp_n(\tau)}{d\tau} = \sum_{m=1}^{\infty} W_{nm} p_m(\tau)$ where p_n is the probability of having n CI molecules/cell. W_{nm} is a matrix coefficient describing the transition rate from state m to state n, and is completely defined by the following 4 rules:

(1) The value of W_{nm} for $n = m + k$ is $a(m) \times g(k; \frac{1}{1+b(m)})$ where $g(k; \frac{1}{1+b(m)}) \equiv \left[\frac{b(m)}{1+b(m)}\right]^k \left[\frac{1}{1+b(m)}\right]$ is a geometric distribution with success probability $\frac{1}{1+b(m)}$. Thus, we interpret the burst size as the number of 'failures' (proteins created) before protein synthesis is successfully turned off (Mehta *et al*, 2008).

(2) The value of W_{nm} for $n = m - 1$ is m. CI is diluted in proportion to growth rate (which is 1 in the units chosen) and copy-number.

(3) The value of W_{nm} for $n < m - 1$ is 0. CI copy-number cannot instantly decrease by more than 1.

(4) In order to conserve probability $W_{nn} \equiv -\sum_{m \neq n} W_{nm}$ i.e. the flux of probability *into all other states* from state m is the flux *out of* m.

By using the experimentally determined burst parameters (see **Estimation of burst parameters from single-cell data**), we were able to fully parameterize the master equation above. The solution of the master equation at arbitrary times was found by numerical matrix exponentiation: $p(n,t) = e^{tW}p(n,0)$, utilizing the **expm** function of Matlab, with a cutoff applied for very high values of n (>2.5x the amount of CI produced per generation if the gene is constantly on). The initial distribution $p(n,0)$ was obtained by evolving a negative binomial distribution for 100 generations, under the initial condition parameters (100% of CI being active.) Finally, in order to determine the fraction of lysogenic cells left at any given time, a threshold value of active CI was chosen based on a value that corresponds to the experimentally chosen partition between states (see **Fig. S4**). The results of this model may be seen in the theoretical predictions (black line and shaded area) of **Fig. 3C-D** and in raw form in **Fig. S7A-B.**

Estimating pleotropic effects of changes in temperature: In analyzing our results, we make the simplifying assumption that the effect of changing temperature is only to vary the fraction of active CI in the cell. Other physiological effects resulting from the change in temperature are neglected. To numerically test the validity of this approximation, we refer to the results of (Herendeen *et al*, 1979), who examined the change in abundance of 100 *E. coli* proteins in the temperature range 13.5-46°C. They found that the majority of proteins measured displayed <20% variation in abundance from 30-42°C, while >95% displayed less than 60% variation. Assuming this is a representative sample, and that this variation is linear in temperature change, we can determine the limits of what is likely to occur in the case of CI (**Fig. S3**). P_{RM} activity was allowed to vary by 30% from the nominal fit (solid line in **Fig. 2A and Fig. S3A**) when temperature is changed from 34°C to 40°C (effectively the worst-case scenario, since the temperature range is ½ that of the 30-42°C range of the study). This variation is shown in the two dashed lines in **Fig. S3A.** This variability was then propagated to the predicted steady states of the system shown in **Fig. 2B** where the dashed lines are again the estimated limits of temperature variation. As seen in **Fig. S3B**, the estimated limits of temperature variation are smaller than the experimental uncertainty in the fit (shaded region in **Fig. 2A-B and Fig. S3A-B.**) In other words, temperature variation is not expected to be a dominant source of error in our analysis.

Friedman N, Cai L & Xie X (2006) Linking Stochastic Dynamics to Population Distribution: An Analytical Framework of Gene Expression. *Physical Review Letters* **97:** 1–4

Gaitanaris GA, Vysokanov A, Hung S-C, Gottesman ME & Gragerov A (1994) Successive action of Escherichia coli chaperones in vivo. *Molecular Microbiology* **14:** 861–869

Herendeen S, VanBogelen R & Neidhardt F (1979) Levels of major proteins of Escherichia coli during growth at different temperatures. *Journal of Bacteriology* **139:** 185–194

Hershey AD (1971) The Bacteriophage lambda New York: Cold Spring Harbor Laboratory

Isaacs FJ, Hasty J, Cantor CR & Collins JJ (2003) Prediction and measurement of an autoregulatory genetic module. *Proceedings of the National Academy of Sciences of the United States of America* **100:** 7714–9

Kampen NG van. (1981) Stochastic processes in physics and chemistry Amsterdam: North-Holland

Levine A, Bailone A & Devoret R (1979) Cellular levels of the prophage lambda and 434 repressors. *Journal of molecular biology* **131:** 655–61

Lieb M (1981) A fine structure map of spontaneous and induced mutations in the lambda repressor gene, including insertions of IS elements. *Molecular & general genetics : MGG* **184:** 364–71

Mehta P, Mukhopadhyay R & Wingreen NS (2008) Exponential sensitivity of noise-driven switching in genetic networks. *Physical biology* **5:** 026005

Reichardt L & Kaiser A (1971) Control of lambda repressor synthesis. *Proceedings of the National …* **68:** 2185–2189

Shahrezaei V & Swain PS (2008) Analytical distributions for stochastic gene expression. *Proceedings of the National Academy of Sciences of the United States of America* **105:** 17256–61

Villaverde A, Benito A, Viaplana E & Cubarsi R (1993) Fine Regulation of c1857-Controlled Gene Expression in Continuous Culture of Recombinant Escherichia coli by Temperature. *Microbiology* **59:** 3485

Zong C, So L, Sepúlveda LA, Skinner SO & Golding I (2010) Lysogen stability is determined by the frequency of activity bursts from the fate-determining gene. *Molecular systems biology* **6:** 440

FIGURE LEGENDS

Table S1: The effect of using different models for the effect of temperature in the *cI857* allele. Our experimental data of P_{RM} activity at different temperatures (**Fig. 2A**) was fitted to a Hill function $f(x) = f_0\{\epsilon + (1-\epsilon)/(1+[x/x_0\,]^{-H}\,)\}$. In doing so, we used four different expressions describing how the fraction of active CI in the cell varies with temperature (the one used in the present study, as well as three from the literature: (Isaacs *et al*, 2003; Zong *et al*, 2010; Villaverde *et al*, 1993)). One sees that the expressions obtained for $f(x)$ are very close to each other. The only exception is that (Zong *et al*, 2010) places the position of half-maximum for the Hill function (x_0) at a significantly lower value than all other models. This difference amounts to a scaling of the assumed number of CI in a lysogen, and reflects the particular way in which the temperature dependence was calibrated based on an earlier study, that of (Schubert *et al*, 2007).

Fig. S1: Single-cell distributions of P_{RM} Activity at different temperatures. Clear bars are the experimentally measured histograms of P_{RM} activity, based on mCherry fluorescence (~100 cells per histogram). Red lines are fits to a gamma distribution (or the sum of two gamma distributions when bimodality was present). The top two sets of panels describe the experiments with initially lysogenic (first row) and initially lytic (second row) cultures. The bottom two sets of panels depict a selection of temperatures zoomed-in by a factor of 10, for clarity.

Fig. S2: Comparison of our results with those of (Schubert *et al*, 2007). **(A)** P_{RM} activity vs. CI concentration for *cro*+ (black squares) and *cro*- (gray squares) reporter strains, reproduced from (Schubert *et al*, 2007). Black line is a fit of the *cro*+ data to a Hill function. Blue circles are the linearly-scaled results of the present study, for comparison. **(B)** Predicted steady-states of P_{RM} promoter activity with respect to the production/elimination ratio. This parameter is analogous to the parameter $\mu(T)$ in our experiments. Note the absence of bistability in the *cro*- strain.

Fig. S3: Estimating pleotropic effects of changes in temperature. **(A)** P_{RM} activity as a function of CI concentration. The main curve, as well as prediction bounds based on experimental error (shaded area), are reproduced from **(Fig. 2A)**. The estimated error due to temperature-dependent variations in protein abundance (see **Estimating pleotropic effects of changes in temperature**) is designated by the dashed lines. **(B)** Predicted steady states of P_{RM} as a function of the fraction of active CI in the cell. The main curve, as well as prediction bounds based on experimental error (shaded area), are reproduced from **(Fig. 2B)**. The estimated error due to temperature-dependent variations in protein abundance (see **Estimating pleotropic effects of changes in temperature**) is designated by the dashed lines.

Fig. S4: Defining the lysis/lysogeny threshold. (A) Experimental fluorescence data from all temperatures shows a clear clustering of cells into subpopulations of lysogenic and lytic cells, predominately along the red and green axes. We therefore classify cells based on a threshold slope in the red/green plane (dashed blue line) **(B)** By determining a threshold angle in the P_R vs. P_{RM} promoter activity plane which effectively separates the two subpopulations experimentally (dashed blue line), we are able to determine a corresponding value of effective CI (dashed red line) which is then used to set the threshold for switching in the stochastic model.

Fig. S5: Fluorescence maturation kinetics. (A) The effect of fluorophore maturation on fluorescence of each channel. Cells from a 30°C-to-38°C temperature shift experiment (see **Fig. 3**) were collected at the 2h time point, resuspended in PBS and stored at 4°C. The fluorescence level was measured at 0, 2 and 12 h after resuspension. Data points are mean over ~100 cells. Error bars are standard error over three independent experiments. **(B)** The mean ratio of green fluorescence to total fluorescence from the same data set as panel A. This ratio was used in the experiment to discriminate lytic from lysogenic cells. The fact that it remains constant between 2 and 12 hours following cell resuspension (when imaging took place) indicates that maturation effects do not bias the measured kinetics.

Fig. S6: **Defining the parameters of the stochastic model. (A)** A Stochastic model for P_{RM} activity. The number of CI molecules per cell (N) increases via random bursts of production (burst size B), and decreases due to cell growth/division. If CI level falls below a threshold (depicted by a wall in the figure), a switch to lysis ensues. **(B)** The average burst size (b) and frequency (a) can be estimated from measuring the mean and variance of CI levels (see **Estimation of burst parameters from single-cell data**) from experimental data at all temperatures. The variance versus mean plot is fitted to a power law, $\sigma^2 = [\frac{f}{\varphi}]^{1+\delta}$, with $\delta = 0.68 \pm 0.14$ and $\varphi = 5.3 \pm 3.5\, generation^{-1}$ in molecular units. The shaded region represents the 95% confidence interval of the fit. **(C)** Estimating the burst parameters. By combining the power-law expression extracted in panel B with the known number of CI molecules in a lysogen (~300) (Reichardt & Kaiser, 1971; Levine *et al*, 1979), we obtain the dependence of burst frequency and size on the number of active CI molecules in the cell. The shaded regions represent error bounds, obtained using 100 random instantiations of fitting parameters, in which both the P_{RM}(CI) and the variance fit of panel B were allowed to vary within their respective confidence intervals. These random instantiations were later used to determine the uncertainty in predicted switching kinetics (see **Fig. 3C-D**.)

Fig. S7: Results of Stochastic Model (A) CI copy-number distribution over time in a simulation of a temperature shift experiment (see **A stochastic model for the lambda switch**.) Cells were initially at the lysogenic state, $\mu = 1$. Results are shown for 4 values of end temperatures, corresponding to $\mu = 0.15, 0.12, 0.09, 0.06$. The left-most panel shows the time evolution over 12 generations following shift to a temperature where both states are still stable. The distribution of CI changes only slightly, due to the very low switching rate. In the second panel, cells are shifted to a higher temperature supporting bistability, and bimodality is observed. In the two rightmost panels, cells are shifted to a temperature in which only lysis is stable, and a fast relaxation to that state is observed. **(B)** The fraction of cells (squares) remaining in the lysogenic state over time for the simulations described in panel **A**. The simulated switching kinetics reproduces the biphasic behavior of the experimental kinetics shown in **Fig. 3B**.

Isaacs FJ, Hasty J, Cantor CR & Collins JJ (2003) Prediction and measurement of an autoregulatory genetic module. *Proceedings of the National Academy of Sciences of the United States of America* **100:** 7714–9

Levine A, Bailone A & Devoret R (1979) Cellular levels of the prophage lambda and 434 repressors. *Journal of molecular biology* **131:** 655–61

Reichardt L & Kaiser A (1971) Control of lambda repressor synthesis. *Proceedings of the National ...* **68:** 2185–2189

Schubert R a, Dodd IB, Egan JB & Shearwin KE (2007) Cro's role in the CI Cro bistable switch is critical for {lambda}'s transition from lysogeny to lytic development. *Genes & development* **21:** 2461–72

Villaverde A, Benito A, Viaplana E & Cubarsi R (1993) Fine Regulation of c1857-Controlled Gene Expression in Continuous Culture of Recombinant Escherichia coli by Temperature. *Microbiology* **59:** 3485

Zong C, So L, Sepúlveda LA, Skinner SO & Golding I (2010) Lysogen stability is determined by the frequency of activity bursts from the fate-determining gene. *Molecular systems biology* **6:** 440

Table S1: Hill fit parameters for various models of temperature dependence

Source	f_0	ε	H	x_0
Present Work	0.77 +/- 0.08	0.033 +/- 0.032	1.80 +/- 0.57	0.062 +/- 0.011
Isaacs et al. 2001	0.75 +/- 0.09	0.032 +/- 0.041	1.83 +/- 0.69	0.054 +/- 0.013
Villaverde et al. 1993	0.75 +/- 0.09	0.032 +/- 0.042	1.86 +/- 0.71	0.047 +/- 0.011
Zong et al. 2010	0.73 +/- 0.08	0.034 +/- 0.043	1.96 +/- 0.73	0.0074 +/- 0.0018

Figure S1.

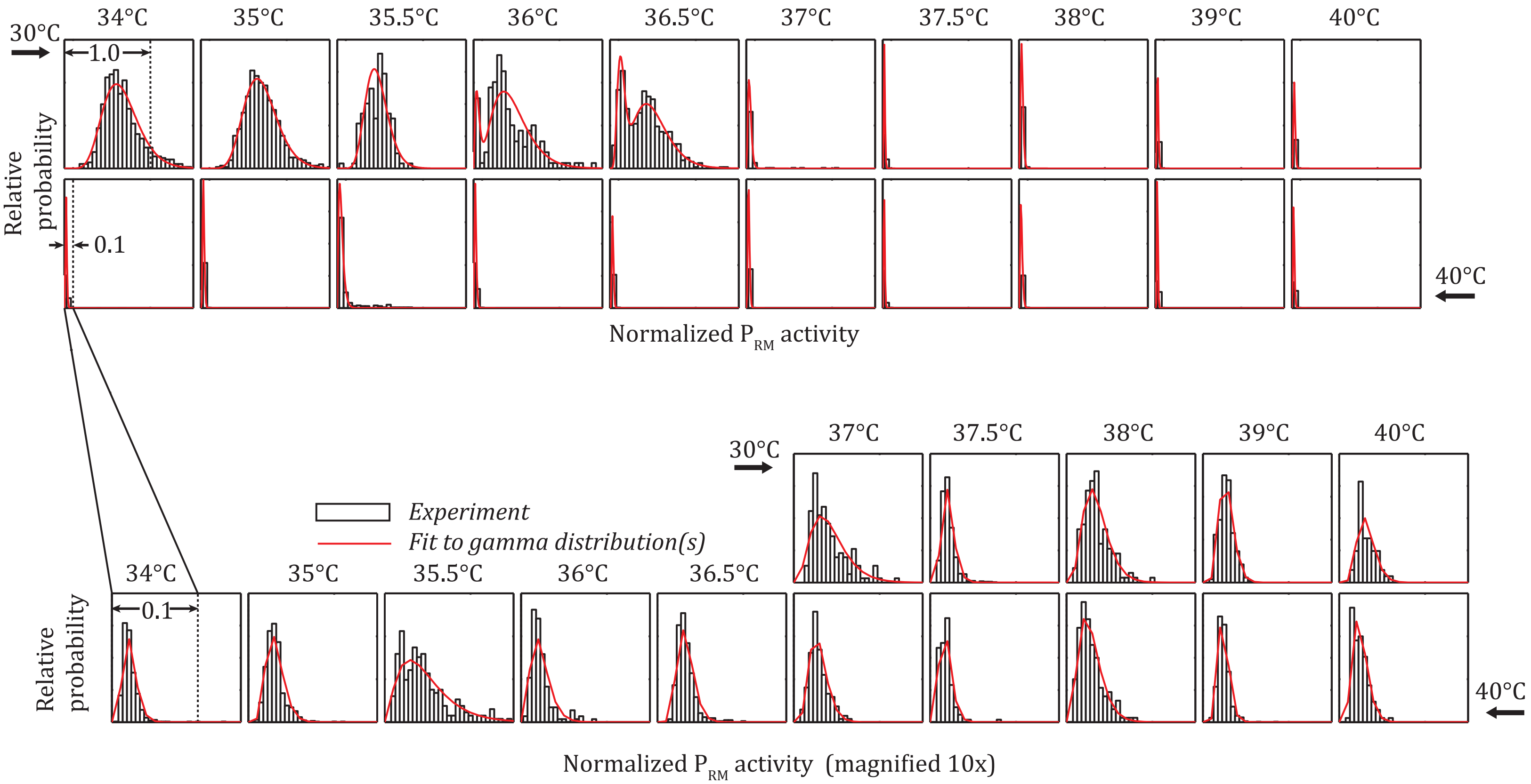

34°C
35°C
35.5°C
36°C
36.5°C
37°C
37.5°C
38°C
39°C
40°C
30°C
1.0
Relative probability
0.1
40°C
Normalized P_{RM} activity
Experiment
Fit to gamma distribution(s)
30°C
37°C
37.5°C
38°C
39°C
40°C
34°C
35°C
35.5°C
36°C
36.5°C
0.1
Relative probability
40°C
Normalized P_{RM} activity (magnified 10x)

Figure S2.

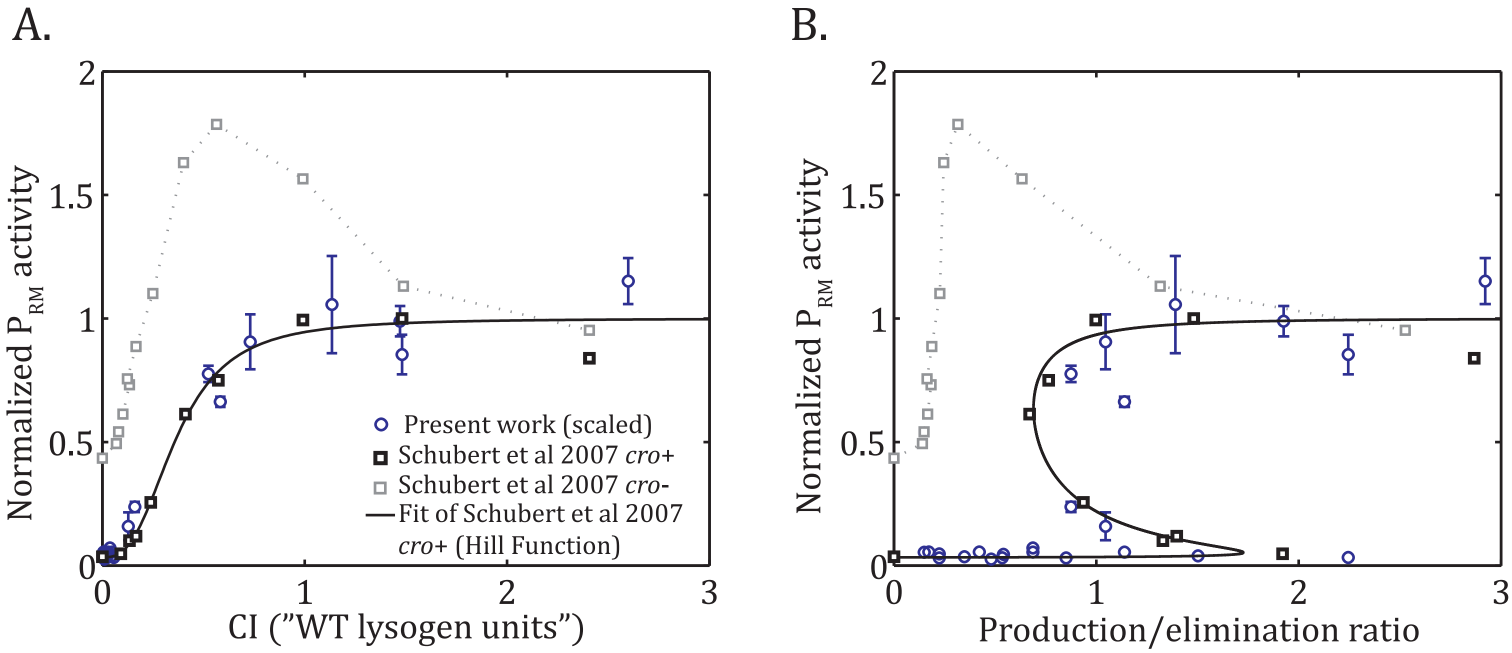

A.
B.
Normalized P RM activity
CI ("WT lysogen units")
Production/elimination ratio
Present work (scaled)
Schubert et al 2007 cro+
Schubert et al 2007 cro-
Fit of Schubert et al 2007
cro+ (Hill Function)

Figure S3.

A.

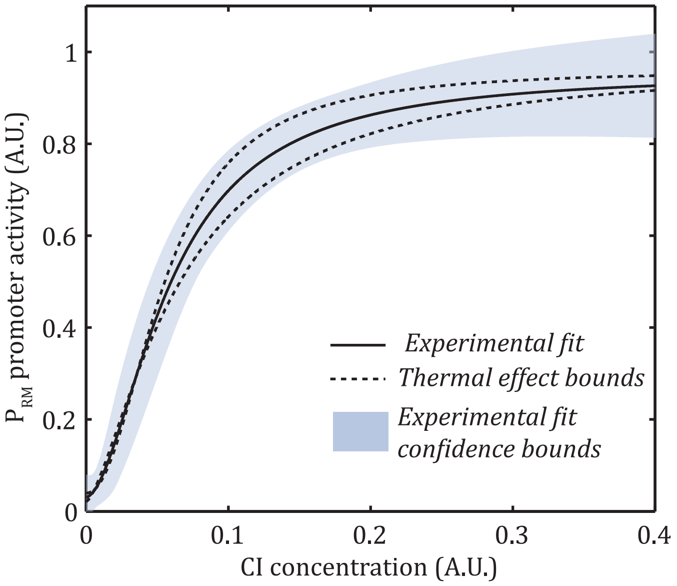

1
0.8
0.6
0.4
0.2
0
0
0.1
0.2
0.3
0.4
P_{RM} promoter activity (A.U.)
CI concentration (A.U.)
Experimental fit
Thermal effect bounds
Experimental fit confidence bounds

B.

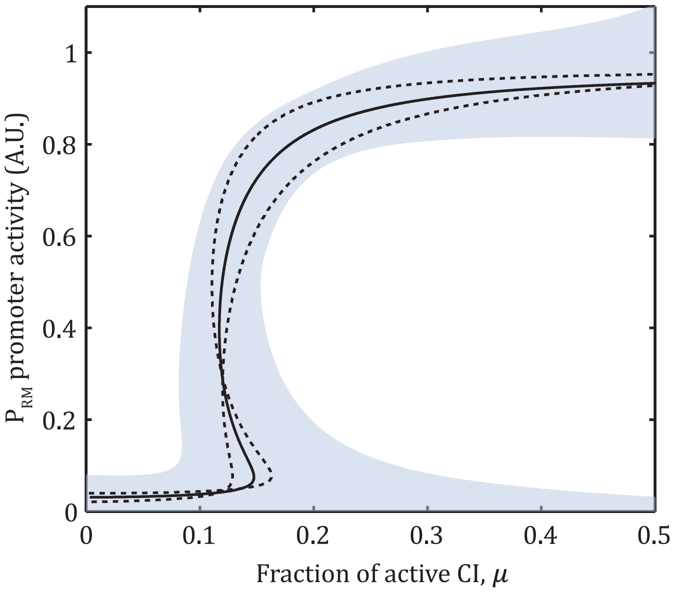

1
0.8
0.6
0.4
0.2
0
0
0.1
0.2
0.3
0.4
0.5
P_{RM} promoter activity (A.U.)
Fraction of active CI, μ

Figure S4.

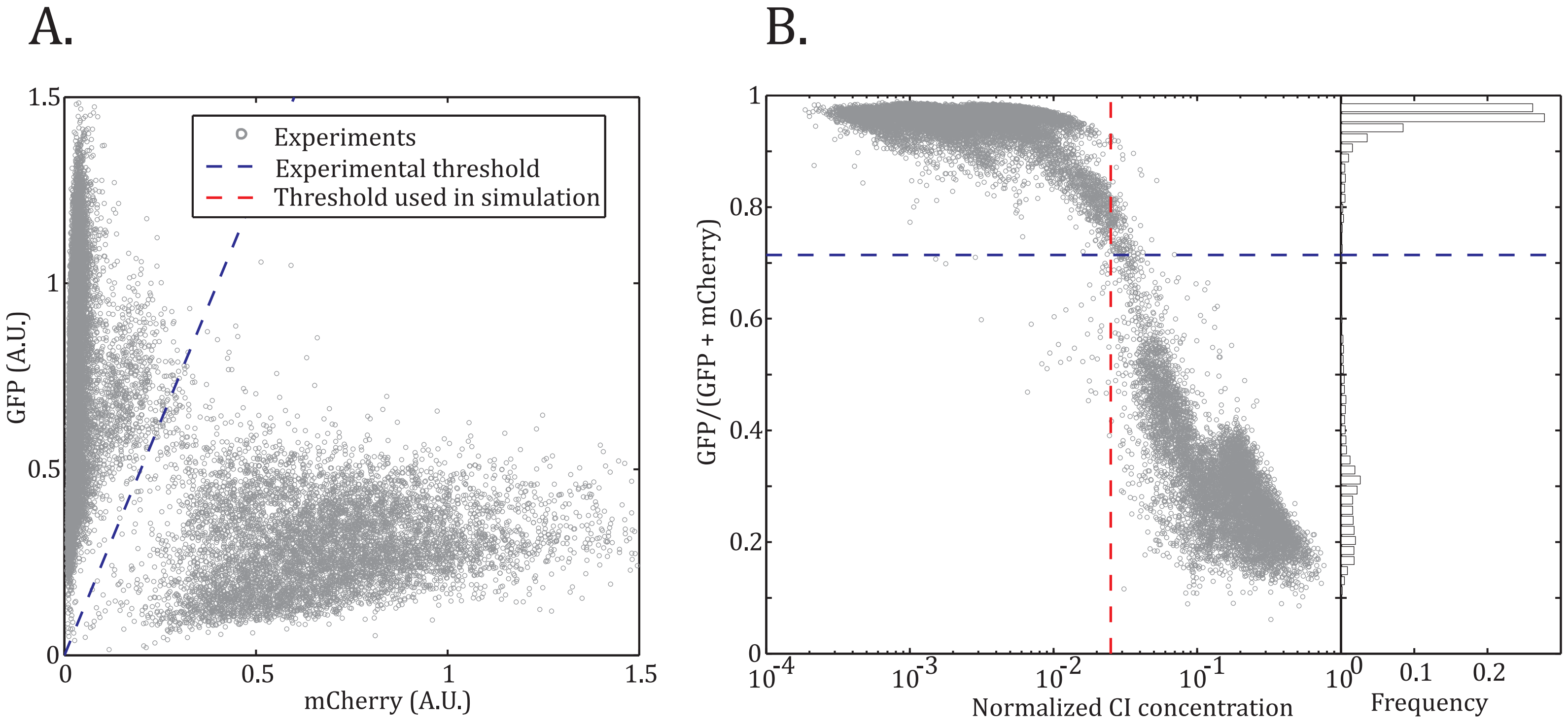

A.
B.
Experiments
Experimental threshold
Threshold used in simulation
GFP (A.U.)
mCherry (A.U.)
0
0.5
1
1.5
GFP/(GFP + mCherry)
0.2
0.4
0.6
0.8
Normalized CI concentration
10^{-4}
10^{-3}
10^{-2}
10^{-1}
10^{0}
Frequency
0.1
0.2

Figure S5.

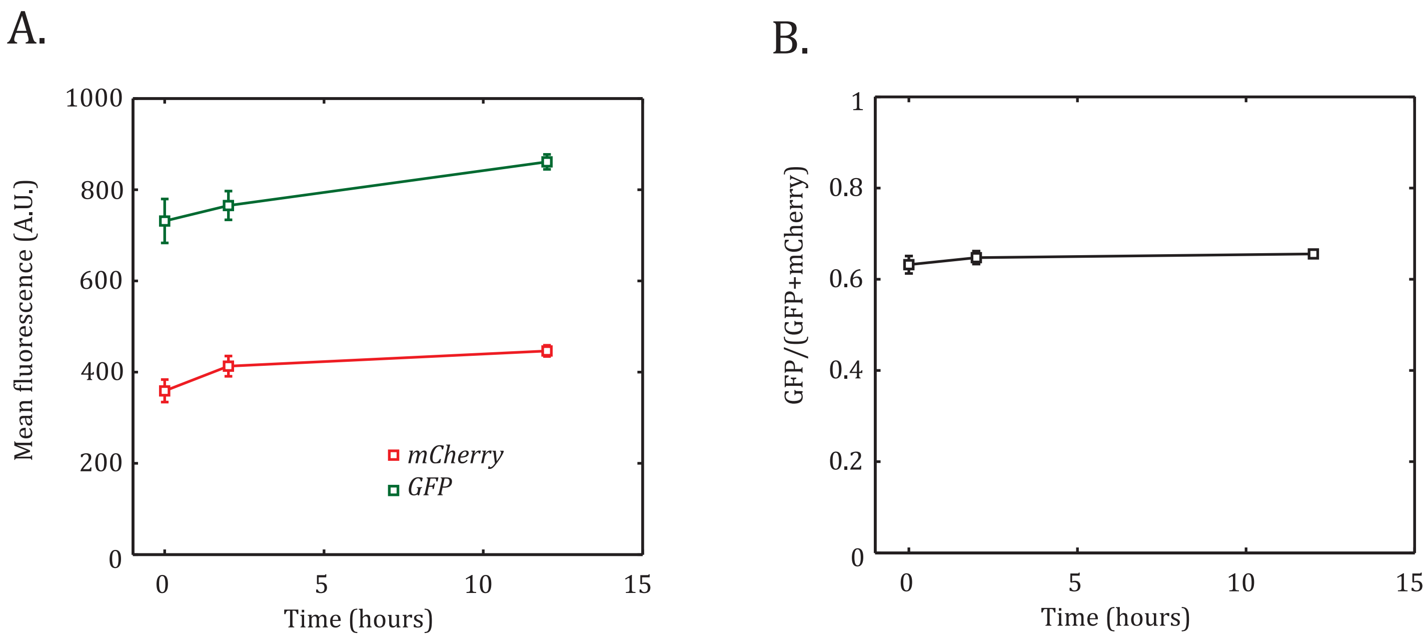
A.
B.
1000
800
600
400
200
0
Mean fluorescence (A.U.)
mCherry
GFP
0
5
10
15
Time (hours)
1
0.8
0.6
0.4
0.2
0
GFP/(GFP+mCherry)
0
5
10
15
Time (hours)

Figure S6.

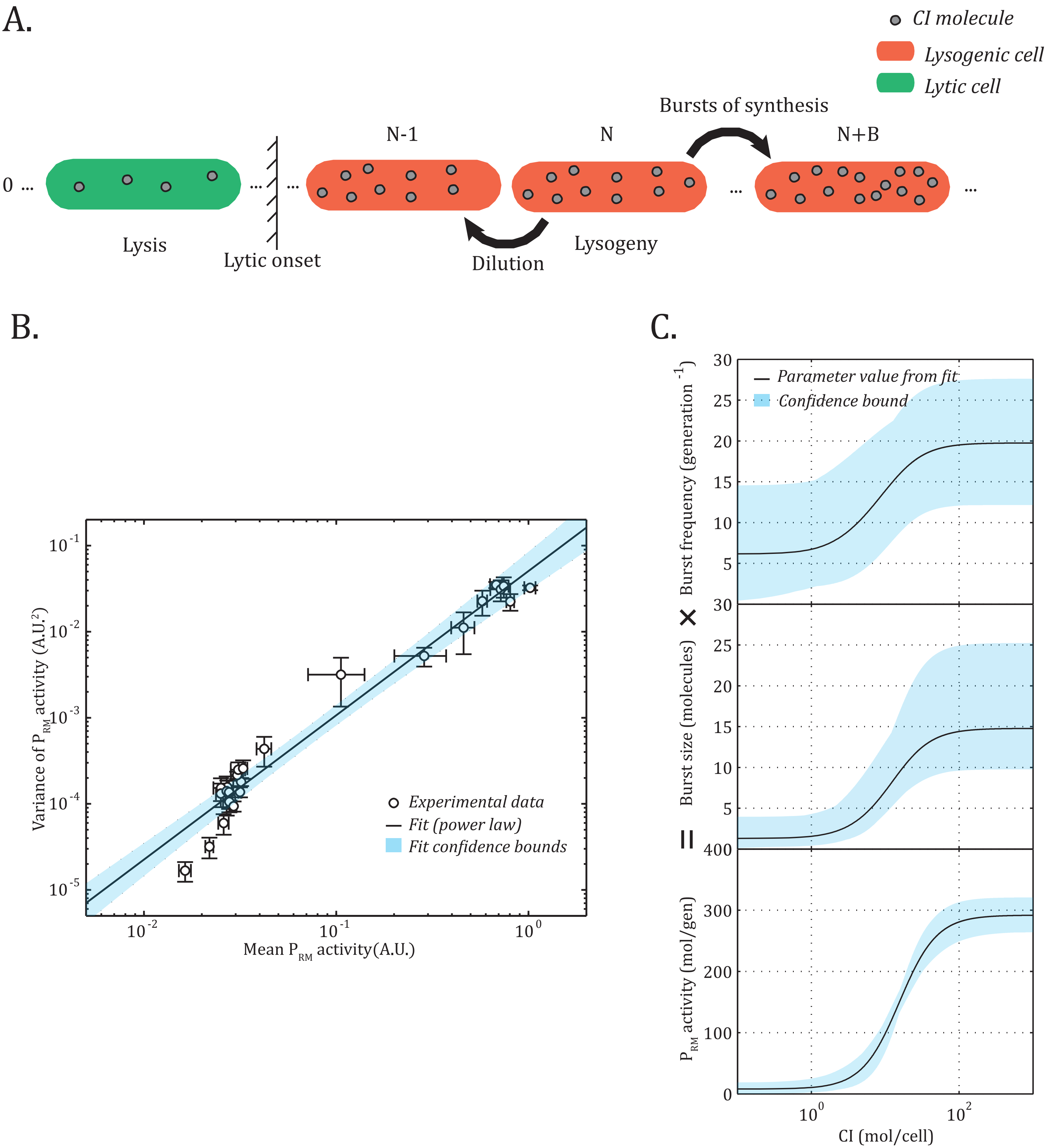

A.
CI molecule
Lysogenic cell
Lytic cell
Bursts of synthesis
N-1
N
N+B
0 ...
...
...
...
...
Lysis
Lytic onset
Dilution
Lysogeny
B.
Variance of P_{RM} activity (A.U.^{2})
Mean P_{RM} activity(A.U.)
10^{-1}
10^{-2}
10^{-3}
10^{-4}
10^{-5}
10^{-2}
10^{-1}
10^{0}
Experimental data
Fit (power law)
Fit confidence bounds
C.
Parameter value from fit
Confidence bound
Burst frequency (generation $^{-1}$)
×
Burst size (molecules)
=
P_{RM} activity (mol/gen)
30
25
20
15
10
5
30
25
20
15
10
5
400
300
200
100
0
10^{0}
10^{2}
CI (mol/cell)

Figure S7

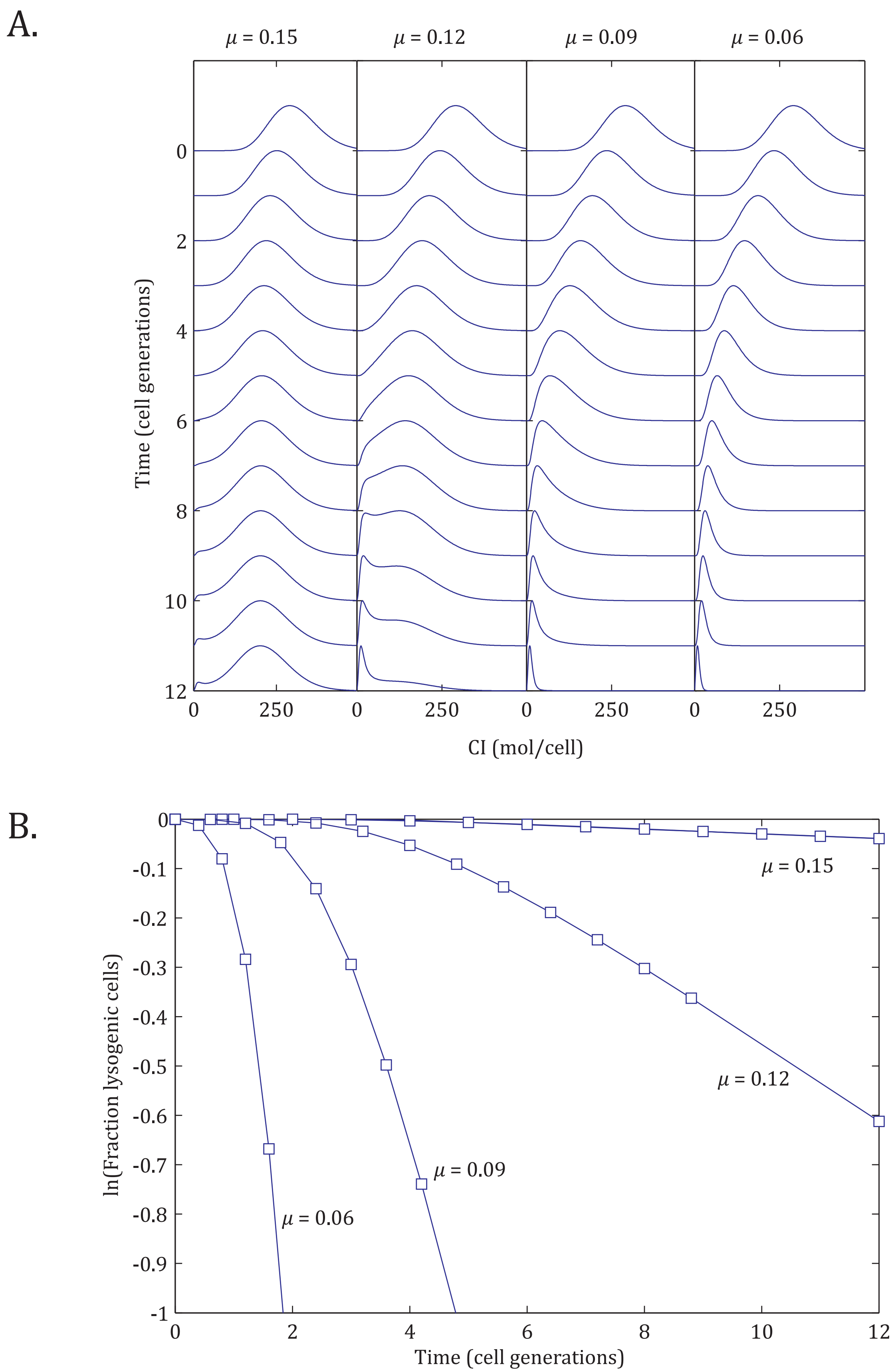
A.
μ = 0.15
μ = 0.12
μ = 0.09
μ = 0.06
Time (cell generations)
0
2
4
6
8
10
12
0
250
CI (mol/cell)
B.
ln(Fraction lysogenic cells)
0
-0.1
-0.2
-0.3
-0.4
-0.5
-0.6
-0.7
-0.8
-0.9
-1
μ = 0.15
μ = 0.12
μ = 0.09
μ = 0.06
Time (cell generations)
0
2
4
6
8
10
12
